# Acid-Sensing Ion Channel 1a Contributes to Airway Hyperreactivity in Mice

**DOI:** 10.1371/journal.pone.0166089

**Published:** 2016-11-07

**Authors:** Leah R. Reznikov, David K. Meyerholz, Ryan J. Adam, Mahmoud Abou Alaiwa, Omar Jaffer, Andrew S. Michalski, Linda S. Powers, Margaret P. Price, David A. Stoltz, Michael J. Welsh

**Affiliations:** 1 Department of Internal Medicine, Roy J and Lucille A Carver College of Medicine, University of Iowa, Iowa City, Iowa, United States of America; 2 Department of Pathology, Roy J and Lucille A Carver College of Medicine, University of Iowa, Iowa City, Iowa, United States of America; 3 Department of Molecular Physiology and Biophysics, Roy J and Lucille A Carver College of Medicine, University of Iowa, Iowa City, Iowa, United States of America; 4 Pappajohn Biomedical Institute, Roy J and Lucille A Carver College of Medicine, University of Iowa, Iowa City, Iowa, United States of America; 5 Department of Biomedical Engineering, College of Engineering, University of Iowa, Iowa City, Iowa, United States of America; 6 Howard Hughes Medical Institute, Roy J and Lucille A Carver College of Medicine, University of Iowa, Iowa City, Iowa, United States of America; Cinvestav-IPN, MEXICO

## Abstract

Neurons innervating the airways contribute to airway hyperreactivity (AHR), a hallmark feature of asthma. Several observations suggested that acid-sensing ion channels (ASICs), neuronal cation channels activated by protons, might contribute to AHR. For example, ASICs are found in vagal sensory neurons that innervate airways, and asthmatic airways can become acidic. Moreover, airway acidification activates ASIC currents and depolarizes neurons innervating airways. We found ASIC1a protein in vagal ganglia neurons, but not airway epithelium or smooth muscle. We induced AHR by sensitizing mice to ovalbumin and found that *ASIC1a*^*-/-*^ mice failed to exhibit AHR despite a robust inflammatory response. Loss of ASIC1a also decreased bronchoalveolar lavage fluid levels of substance P, a sensory neuropeptide secreted from vagal sensory neurons that contributes to AHR. These findings suggest that ASIC1a is an important mediator of AHR and raise the possibility that inhibiting ASIC channels might be beneficial in asthma.

## Introduction

Studies over the last few decades indicate that the nervous system is a critical mediator of hallmark features of asthma, including cough, mucus secretion and airway hyperreactivity (AHR) [1–6]. Several lines of evidence support this conclusion. First, greater sensory nerve innervation [7] and increased levels of sensory neuropeptides have been observed in asthmatic airways [8, 9]. Second, persistent AHR in the absence of inflammation is associated with a doubling of airway smooth muscle innervation [10]. Third, ablation of airway sensory neurons that express the *transient receptor potential vanilloid 1* (*TRPV1*) gene reduces AHR in inflamed airways [11]. Fourth, inactivation of sensory neurons expressing Nav1.8 or blockade of Nav1.8 reduces AHR and asthmatic symptoms [12, 13]. Fifth, elimination of the transient receptor potential cation channel, subfamily A, member 1 (TRPA1), a sensory neuron receptor, decreases AHR [14, 15]. Sixth, acute vagotomy just prior to methacholine challenge prevents AHR in mice [16]. Seventh, anticholinergics and sympathomimetics are neurotransmitter antagonists and agonists that mitigate bronchoconstriction [17, 18] and AHR [19, 20].

Both nociceptors and non-nociceptors innervating the airway express Nav1.8 [21], and approximately 50% of Nav1.8-positive neurons express TRPV1 [12]. Many TRPV1-positive neurons innervating the airway also express TRPA1 [22]. Eliminating neurons that expressed TRPV1 prevented AHR in a murine model of asthma [11], without decreasing inflammation. In contrast, silencing neurons that express Nav1.8 [12] or elimination of TRPA1 prevented AHR [14] and decreased inflammation. These findings suggest that a unique subset of vagal ganglia neurons modifies airway inflammation and AHR, whereas another subset, characterized by the expression of TRPV1, modifies only the manifestation of AHR. Since loss of the *TRPV1* gene itself did not protect against AHR [14], then it is unlikely that TRPV1 is the key sensor that mediates AHR. It also seems unlikely that TRPA1 is key receptor in TRPV1-expressing neurons since eliminating TRPA1 decreases inflammation [14], yet inflammation remained unchanged in mice with selective ablation of TRPV1-expressing neurons [11]. Thus, the sensor mediating AHR in TRPV1-expressing neurons remains uncertain.

In addition to expressing TRPA1[22], vagal airway sensory neurons that express TRPV1 also express acid-sensing ion channels (ASICs) [23–28]. ASICs are voltage-insensitive cation channels in the epithelial Na^+^ channel/degenerin superfamily that are activated by extracellular protons [29, 30]. Several studies indicate that the airway becomes acidic in asthma [31–34]. The proposed mechanisms inducing acidification are immune cell infiltration, inflammation and oxidative stress [34]. Of note, aspiration can also acidify the airways and elicit asthmatic symptoms [35, 36]. In addition, acid inhalation and airway acidification induce airway constriction [37–40]. The airway acidification elicits airway constriction through activation of TRPV1 and ASICs, and the subsequent release of sensory neuropeptides such as tachykinins [24, 27, 37–41]. Therefore, ASICs might play a key role in mediating AHR.

Rat vagal airway sensory neurons express *ASIC1a*, *-1b*, *-2* and *-3* mRNA [24]. Approximately 45% of rat vagal airway sensory neurons display H^+^-gated currents with the features of both TRPV1 and ASIC channels [23]. The transient component of those H^+^-gated currents has properties characteristic of ASIC currents and is blocked by the ASIC blocker amiloride [23]. The sustained component has properties of TRPV1 currents and is blocked by the TRPV1 antagonist capsazepine. The onset of acid-evoked action potentials in airway vagal sensory neurons coincides with ASIC-mediated depolarization, but not TRPV1-mediated depolarization [23]. From those studies, the authors concluded that ASIC1, -2, and -3 are responsible for the ASIC currents in rat airway vagal sensory neurons. A separate study supported that conclusion and found that ASIC currents in rat airway vagal sensory neurons were likely due to heteromers consisting of some combination of ASIC1, -2 and -3 [26]. Application of acid to guinea pig vagal nerve fibers innervating the airway also elicited currents with characteristic properties of ASIC channels [28]. Of note, some airway vagal sensory neurons in the guinea pig demonstrated H^+^-gated currents consistent with expression of only ASICs, and not both ASICs and TRPV1 [28]. A similar finding has been found in vagal neurons innervating the esophagus, where mRNA expression of ASIC1, 2, and 3 is found in TRPV1-negative neurons [42]. Collectively, the location and function of ASICs suggests that they might be important mediators of AHR.

To study whether ASICs channels modified AHR, we studied *ASIC1a*^*-/-*^ mice. Previous work showed that disrupting the *ASIC1a* gene modifies the physiological properties of H^+^-gated currents in neurons and behavioral responses to acid [43, 44]. We did not study *ASIC2*^*-/-*^ mice because they exhibit an impaired baroreceptor reflex [45], which could affect tracheal dilation [46]. We also did not study *ASIC3*^*-/-*^ mice because they have diminished sympathetic tone [47], which could confound airway resistance measurements. We did not use a pharmacological approach because the mixed pharmacology and state-dependent activity of many ASIC channel blockers, such as amiloride [48], PcTx1[49, 50], APETx2 [51, 52], and Diclofenac [53] would make interpretation of results less clear.

## Materials and Methods

### Animals

Adult (8–9 weeks old) A*SIC1a*^*-/-*^ [54] and wild-type male mice were maintained on a congenic C57BL/6J background. These studies were approved by the University of Iowa Animal Care and Use Committee.

### OVA sensitization

Mice were sensitized as previously described [55, 56]. Briefly, 8–9 week-old mice were sensitized by intraperitoneal injection of 10 μg of OVA (Sigma) mixed with 1 mg of alum in 0.9% saline on days 0 and 7. Control mice received saline with 1 mg of alum on days 0 and 7. On days 14–16, mice received 1% OVA or 0.9% saline for 40 min by nebulization.

### Bronchoalveolar lavage and analyses

All mice that completed FlexiVent procedures were subjected to a bronchoalveolar lavage. Lungs received three sequential 1 ml lavages of 0.9% sterile saline delivered into the airways through a cannula secured in the euthanized mouse trachea. All collected material from one mouse was pooled, spun at 500 X g, and the supernatant was removed and frozen at -80°C.

Cell count analysis and percent granulocytes was calculated once as previously described [57]. IL4, IL5, and IL13 were assayed by DuoSet ELISA kits (R&D Systems). Each ELISA was run once; duplicates of the lavage fluid per each animal were run. Substance P was assayed by ELISA (Enzo Life Sciences) and performed after cytokines were assessed. Because of variations in the amount of retrieved bronchoalveolar lavage fluid, adequate amounts of bronchoalveolar lavage fluid were only available from 6 wild-type non-sensitized mice, 6 wild-type OVA-sensitized mice, 6 A*SIC1a*^*-/-*^ non-sensitized mice, and 7 A*SIC1a*^*-/-*^ OVA-sensitized mice. Duplicates of the lavage fluid per each animal were run. All ELISAs were performed according to the manufacturer’s instructions.

### Vagal ganglia isolation

Mice were euthanized by overdose of isofluorane inhalation. The vagal ganglia were exposed by gently pulling on the vagus nerve and then delicately cutting. They were immediately placed in RIPA buffer and stored at -80°C until protein isolation.

### Western blot

Total protein from mouse brain, vagal ganglia, trachea, and lung were isolated using RIPA buffer (Sigma). Samples (40 μg) were denatured and run on a 4–15% polyacrylamide gel. Whole brain lysate from a wild-type mouse served as a control (10 μg loaded). A rabbit polyclonal antisera directed against mouse ASIC1a was provided as a kind gift from Dr. John Wemmie for western blot analysis. The western blotting of vagal ganglia was performed on two separate occasions using pooled tissues from the same three wild-type mice; similar results were observed. The airway was assessed by western blot on three separate occasions using pooled tissues from three wild-type mice; similar results were obtained.

### Immunocytochemistry

Whole vagal ganglia were dissected from wild-type and *ASIC1a*^*-/-*^ mice and fixed in 2% PFA for 15 min. Samples were then washed and permeabilized as previously described [58]. Samples were incubated in anti-ASIC1a polyclonal goat antibody (Sigma) at a ratio of 1:250 overnight at room temperature with gentle shaking. An alexa 488 secondary antibody (Life Technologies) at 1:500 was used for detection. Sections were mounted with vectashield and viewed with an Olympus Fluoview confocal microscope. Images were taken with identical settings. Post-collection adjustments were made identically. Two vagal ganglia from one wild-type and one A*SIC1a*^*-/-*^ mouse were assessed by immunocytochemistry; similar results were achieved. The airway was assessed by immunocytochemistry on four separate occasions using independent wild-type and A*SIC1a*^*-/-*^ mice; similar results were achieved.

### Quantitative RT-PCR

RNA from total mouse airways and vagal ganglia was isolated using Qiagen Lipid Kit and treated with DNAse. RNA integrity was assessed by an Agilent Bioanalyzer. RNA was then reverse transcribed using VILO mastermix. Primers were designed for murine *muc5AC* as previously described [55]. Transcript abundance was assessed once. RNA was isolated from the airways of 8 wild-type non-sensitized mice, 7 wild-type OVA-sensitized mice, 7 A*SIC1a*^*-/-*^ non-sensitized mice, and 7 A*SIC1a*^*-/-*^ OVA-sensitized mice.

### Mouse cultures

Mouse tracheal epithelial cells were cultured as previously described [59].

### FlexiVent

Flexivent experiments were carried out on two separate cohorts of mice. Ketamine and xylazine sedation were used to preserve vagal reflexes [60, 61]. For each cohort, one mouse from each genotype and treatment was run on a single day. Data were collected over a period of 4 days for each cohort. FlexiVent procedures were performed as previously described [55]. Increasing doses of methacholine were aerosolized using an ultrasonic nebulizer. The aerosols were delivered for 10 sec into the inspiratory line of the ventilator. Measurements for each methacholine dose were taken at 10 sec intervals over the course of 5 mins. Two wild-type OVA-sensitized mice died during FlexiVent procedures (one from each cohort) and their FlexiVent data were not used. One wild-type non-sensitized mouse died during tracheostomy and was not included in the study.

### Chemicals

Acetyl-beta-methacholine-chloride (Sigma) was dissolved in 0.9% saline for flexiVent studies.

### Histopathology

Following euthanasia, the left lung was removed and placed in 10% normal buffered formalin. Lungs were removed from all animals that underwent OVA-sensitization and their respective non-sensitized controls. A single wild-type OVA-sensitized mouse lung was not collected due to user error. Samples were sectioned and stained as previously described [62]. A pathologist masked to groups performed scoring on H&E stained mouse lung sections [63]. The following scores were assigned for bronchovascular inflammation severity: 1, within normal limits; 2, focal solitary cells with uncommon aggregates; 3, multifocal nominal to moderate sized aggregates; 4, moderate to high cellularity, multifocal large cellular aggregates that may be expansive into adjacent tissues. The following scores were assigned for bronchovascular inflammation distribution: 1, within normal limits; 2, minor to localized aggregates, <33% of lung; 3, multifocal aggregates, 33–66% of lung; 4, coalescing to widespread, >66% of lung. Scoring occurred once.

### Lung fixation and micro-CT scanning

Mice were euthanized with an intraperitoneal injection (Euthasol; Vibrac, Fort Worth, TX), and their lungs were surgically excised. The lungs were fixed via airway instillation at a pressure of 25 cmH_2_O as previously described [64]. The fixative was composed (by volume) of 55% distilled water, 25% polyethylene glycol, 10% ethyl alcohol (190 proof), and 10% formaldehyde. The lungs were removed from the fixative after 24 hr and placed in an oven at 60°C for 24 hr. While in the oven, an airway pressure of 25 cmH_2_O was maintained.

Lungs were imaged by micro computed tomography (micro-CT) on a Siemen’s Inveon PET/CT/SPECT scanner. Scanner settings were: 50 kVp voltage, 500 μA current, 2150 ms exposure time, 360 degrees of rotation, and 720 projections. The resulting voxels were cuboidal with 40 μm sides. Airway measurements were obtained from the micro-CT scans with Pulmonary Workstation 2.0 (VIDA Diagnostics Inc., Coralville, IA) as previously described [64]. Measurements were made perpendicular to the airway centerline and were obtained for the 35 airways highlighted by Thiesse *et al*. [65]. Airway measurements occurred over a period of several days, with the operator blinded to genotype.

### Statistical analysis

We designed our study based upon an anticipated effect size of 1.6–1.8. These values were obtained from previous data generated from published literature [55]. Using a g-power analysis for a two-tailed “Difference between two independent means (two groups)”, the calculated number of animals required for an alpha value of 0.05 and a beta value of 0.2 was 6–8 animals per group. A two-way ANOVA was performed for studies with two or more groups and two or more conditions. When two or more groups were present, but only one condition was being tested, a one-way ANOVA was performed. Post-hoc comparisons were performed using a LSD test. For micro-CT studies, a test of normality was performed indicating data was not normally distributed. Therefore, a Mann Whitney test was used to assess differences on the combined total airway lumen area (35 segmented branches combined for each genotype). For histopathological scoring, a non-parametric ANOVA was used; when significance was found, non-parametric a Mann Whitney test between two individual groups was performed. Significance for all tests was assessed as p<0.05. Exact p values are shown in figure legends.

## Results

**Fig 1:**
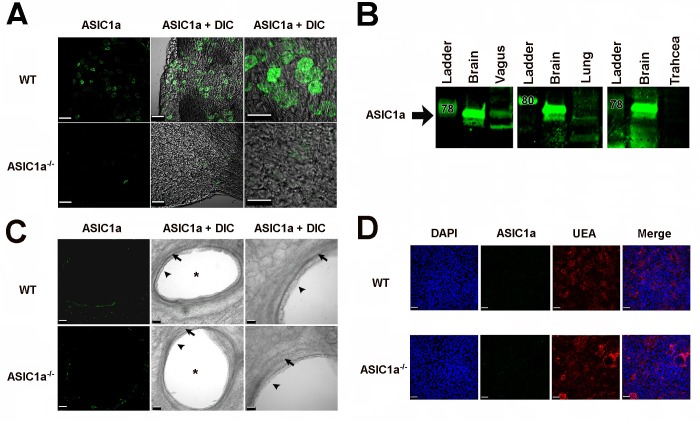
ASIC1a is present in vagal ganglia and expression in the airway is non-specific or negligible. **A)** Images of wild-type (WT) and *ASIC1a*^*-/-*^ mouse vagal ganglia. ASIC1a immunostaining is in green, and DIC indicates differential interference contrast images. Scale bar in left and middle panels is 60 μm; scale bar in the right panel is 40 μm. **B)** Western blot of ASIC1a in the vagal ganglia. Brain is a positive control. For vagal ganglia, trachea, and lung, 40 μg of protein from tissues of 3 WT mice were pooled. For brain, 10 μg of protein was loaded. **C**) Images of wild-type (WT) and *ASIC1a*^*-/-*^ mouse lung cross-sections. ASIC1a immunostaining is shown in green, and DIC indicates differential interference contrast images. Scale bar in left and middle panels is 50 μm; scale bar in the right panel is 30 μm. Asterisks indicate airways; arrowheads show epithelia; arrows identify smooth muscle. **D**) Images of wild-type (WT) and *ASIC1a*^*-/-*^ mouse cultured airway epithelia. DAPI staining is blue (nuclei), ASIC1a immunostaining is in green, ulex europaeus agglutinin (UEA) staining is red (mucin-producing cells), and DIC indicates differential interference contrast images. Scale bar is 30 μm. Abbreviations: WT, wild-type; ASIC, acid-sensing ion channel; DIC, differential interference contrast. UEA, ulex europaeus agglutinin; DAPI, 4',6-diamidino-2-phenylindole. Staining of airways and cultures occurred using same procedures and same conditions as the vagal ganglia.

Immunofluorescence and western blotting revealed ASIC1a expression in the vagal ganglia, consistent with earlier work [24] (Fig 1A and 1B). We found negligible protein expression in the lung (Fig 1B). Compared to *ASIC1a*^*-/-*^ tissue, tissue from wild-type mice showed no specific immunostaining in airway smooth muscle (Fig 1C) or airway epithelia (Fig 1C and 1D), even though immunostaining procedures occurred at the same time and under the same conditions as the vagal ganglia immunostaining. We made numerous attempts to identify ASIC1a immunostaining in nerve endings innervating the airway, but no specific staining was observed.

**Fig 2:**
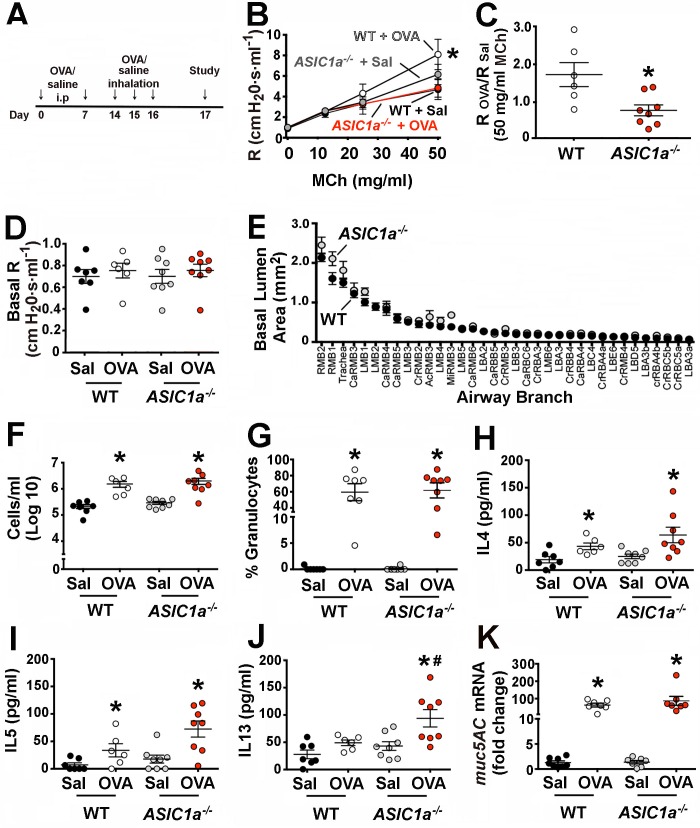
Loss of ASIC1a prevents airway hyperreactivity. **A)** Male mice (8–9 weeks-old) were sensitized by intraperitoneal injection of 10 μg of OVA (Sigma) mixed with 1 mg of alum in 0.9% saline on days 0 and 7. Control mice received saline with 1 mg of alum at day 0 and 7. On days 14–16, mice were nebulized with either 1% OVA or 0.9% saline for 40 min in a chamber. **B)** Airway resistance (R) was measured by flexiVent in OVA-sensitized wild-type and *ASIC1a*^*-/-*^ mice before and following administration of increasing doses of methacholine. Data are mean±SEM. WT + Sal, n = 7 mice; WT + OVA, n = 6 mice; *ASIC1a*^*-/-*^ + Sal, n = 8 mice; *ASIC1a*^*-/-*^ + OVA, n = 8 mice. * indicates p = 0.043. **C)** Ratio of airway resistance after administration of 50 mg/ml methacholine in OVA-sensitized mice compared to non-sensitized mice. A ratio of 1 indicates that airway resistance of OVA-sensitized and non-sensitized mice was the same. * indicates p = 0.012. Ratios for *ASIC1a*^*-/-*^ mice were not statistically different from one (p = 0.18). **D)** Baseline airway resistance (R) prior to administering methacholine. p = 0.89. **E)** Airway measurements obtained from micro-CT scans. Data are mean±SEM area for 35 different airways. Airways are shown according to size. References to abbreviations and methods are in the Methods section. WT, n = 4 mice; *ASIC1a*^*-/-*^, n = 5 mice. p = 0.35. **F**) Number of cells in bronchoalveolar lavage fluid from non-sensitized and sensitized mice. For WT + Sal vs. WT + OVA, * indicates p = 0.004; for *ASIC1a*^*-/-*^ + Sal vs. *ASIC1a*^*-/-*^ + OVA, * indicates p = 0.006. **G)** The percentage of granulocytes in bronchoalveolar lavage fluid. For WT + Sal vs. WT + OVA, * indicates p<0.0001; for *ASIC1a*^*-/-*^ + Sal vs. *ASIC1a*^*-/-*^ + OVA, * indicates p<0.0001. **H**) Levels of IL4 in bronchoalveolar lavage fluid. For WT + Sal vs. WT + OVA, * indicates p = 0.03; for *ASIC1a*^*-/-*^ + Sal vs. *ASIC1a*^*-/-*^ + OVA, * indicates p = 0.018. **I)** Levels of IL5 in bronchoalveolar lavage fluid. For WT + Sal vs. WT + OVA, * indicates p = 0.049; for *ASIC1a*^*-/-*^ + Sal vs. *ASIC1a*^*-/-*^ + OVA, * indicates p = 0.005. A Pearson’s normality test showed that IL5 values in the *ASIC1a*^*-/-*^ OVA-sensitized mice do not differ from a normal distribution. **(J)** Levels of IL13 in bronchoalveolar lavage fluid. For WT + Sal vs. WT + OVA, p = 0.054; for *ASIC1a*^*-/-*^ + Sal vs. *ASIC1a*^*-/-*^ + OVA, * indicates p = 0.013; for WT + OVA vs. *ASIC1a*^*-/-*^ + OVA, # indicates p = 0.036. A Pearson’s normality test showed that IL13 values in the *ASIC1a*^*-/-*^ OVA-sensitized mice do not differ from a normal distribution. **K**) *muc5AC* mRNA in mouse airways. For WT + Sal vs. WT + OVA, * indicates p<0.0001; for *ASIC1a*^*-/-*^ + Sal vs. *ASIC1a*^*-/-*^ + OVA, * indicates p = 0.018. For all panels, individual points represent data collected from a single mouse. Bars and whiskers indicate mean±SEM. Abbreviations: OVA, ovalbumin; Sal, saline; WT, wild-type; ASIC, acid-sensing ion channel; MCh, methacholine.

We induced AHR by using a common sensitization protocol that elicits an allergic reaction to ovalbumin (OVA) [55, 56]. Briefly, adult mice received OVA intraperitoneally on days 0 and 7 (Fig 2A). On days 14–16, mice inhaled a 1% OVA/saline solution to elicit an airway-specific reaction. On day 17, we assessed AHR by measuring airway resistance in response to nebulized methacholine; in mice that exhibit AHR, the degree of airway narrowing in response to methacholine is exaggerated and reflected as a higher airway resistance. As the concentration of methacholine increases, the degree of airway narrowing increases and thus airway resistance increases. Both non-sensitized wild-type and *ASIC1a*^*-/-*^ mice showed similar airway resistance in response to increasing concentrations of methacholine (Fig 2B). As expected, OVA-sensitization induced AHR in wild-type mice (Fig 2B and 2C). The degree of AHR was consistent with previous reports in C57Bl/6 mice [55, 66]. In contrast, OVA-sensitization failed to elicit AHR in *ASIC1a*^*-/-*^ mice.

One possible explanation for the lack of AHR in *ASIC1a*^*-/-*^ mice might be that *ASIC1a*^*-/-*^ mice contained larger airways. However, baseline airway resistance was not reduced in the *ASIC1a*^*-/-*^ mice (Fig 2D). In addition, micro-CT studies and airway segmentation analysis revealed no differences in the airway lumen diameters between genotypes (Fig 2E). Thus, a larger airway diameter did not explain the lack of AHR in *ASIC1a*^*-/-*^ mice.

**Fig 3:**
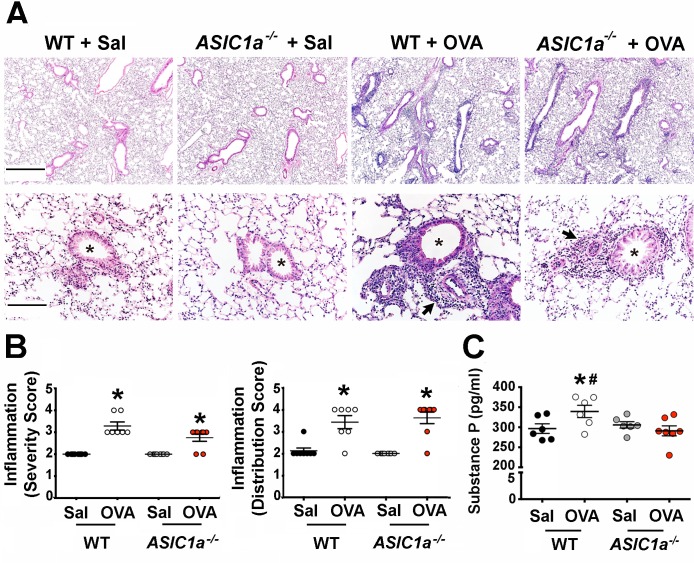
OVA-sensitization induces similar bronchovascular inflammation in wild-type and *ASIC1a*^*-/-*^ mice. **A)** Representative hematoxylin and eosin staining of mouse lung sections. Asterisks indicate airways; arrows indicate examples of bronchovascular inflammation. Scale bar for top panels indicates 700 μm; for lower panels bar indicates 140 μm. **B)** Bronchovascular inflammation score. Bronchovascular inflammation severity was scored as follows: 1, within normal limits; 2, focal solitary cells with uncommon aggregates; 3, multifocal nominal to moderate sized aggregates; 4, moderate to high cellularity, multifocal large cellular aggregates that may be expansive into adjacent tissues. The following scores were assigned for bronchovascular inflammation distribution: 1, within normal limits; 2, minor to localized aggregates, <33% of lung; 3, multifocal aggregates, 33–66% of lung; 4, aggregates coalescing to widespread, >66% of lung. For severity score: WT + Sal vs. WT + OVA, * indicates p = 0.0002; for *ASIC1a*^*-/-*^ + Sal vs. *ASIC1a*^*-/-*^ + OVA, * indicates p = 0.007. For distribution score: WT + Sal vs. WT + OVA, * indicates p = 0.005; for *ASIC1a*^*-/-*^ + Sal vs. *ASIC1a*^*-/-*^ + OVA, * indicates p = 0.001. **C)** Substance P measured by ELISA in the bronchoalveolar lavage fluid as a test of sensory nerve activity. For WT + Sal vs. WT + OVA, * indicates p = 0.05; for WT + OVA vs. *ASIC1a*^*-/-*^ + OVA, # indicates p = 0.03. For panels B and C, each symbol indicates data from an individual mouse. Bars and whiskers indicate mean±SEM. Abbreviations: OVA, ovalbumin; Sal, saline; WT, wild-type; ASIC, acid-sensing ion channel.

Airway inflammation is a key component of asthma and of the OVA-sensitization model [67, 68] and is characterized by the presence of granulocytes and increased levels of Th2 cytokines such as IL13, IL4 and IL5 [69–71]. Accordingly, we asked whether *ASIC1a* gene disruption reduced the inflammatory response to OVA. As previously reported [55], OVA-sensitization induced inflammatory cells in the bronchoalveolar lavage fluid of wild-type mice. OVA-sensitization *ASIC1a*^*-/-*^ mice also showed increased inflammatory cells in the bronchoalveolar lavage fluid (Fig 2F and 2G). Both genotypes of mice showed a similar induction of key inflammatory cytokines in response to OVA-sensitization (Fig 2H–2J), although IL13 levels were unexpectedly statistically greater in OVA-sensitized *ASIC1a*^*-/-*^ mice than in controls. Inflammation increases mucus production, and as such, increased mucus is a manifestation of asthma and inflammation [72]. We found that OVA-sensitization increased transcripts for *muc5AC*, the major murine airway mucin glycoprotein in mucus, in both wild-type and *ASIC1a*^*-/-*^ mice (Fig 2K). This result was consistent with the presence of robust inflammation in both genotypes. We also assessed inflammation using quantitative histopathology. As expected and consistent with our other findings, OVA-sensitization increased bronchovascular inflammation in both wild-type and *ASIC1a*^*-/-*^ mice (Fig 3A). The severity and distribution of bronchovascular inflammation did not differ between genotypes (Fig 3B). Collectively, these findings suggest that loss of ASIC1a decreases AHR without reducing the inflammatory response.

Substance P is a tachykinin mediator of airway smooth muscle contraction in many species, including mice [73, 74] and contributes to AHR [15, 75–77]. Substance P is enriched in c-fibers and its release is thought to reflect c-fiber activity [78, 79]. Consistent with this, disrupting sensory nerve function by eliminating the TRPA1 chemosensory receptor decreases substance P and other sensory neuropeptides in the bronchoalveolar lavage fluid of mice [14]. Therefore, we used substance P as an indicator of sensory nerve function. We measured the concentration of substance P in the bronchoalveolar lavage fluid and found that loss of ASIC1a prevented the OVA-induced increase in substance P (Fig 3C). This result suggests that part of the protection against AHR might involve reduced sensory nerve function and/or decreased release of pro-contractile neuropeptides, such as substance P.

## Discussion

Our data show that disrupting the *ASIC1a* gene prevented AHR in an OVA-sensitization model. They also emphasize the importance of the nervous system in the manifestation of AHR.

Although inflammation is a prerequisite for the development of AHR in allergic asthma [68], we found that loss of ASIC1a decreased AHR without reducing inflammation. That dissociation has also been reported by others. For example, Trankner and colleagues ablated a population of vagal sensory neurons in mice and found that it prevented AHR following OVA-sensitization, but did not reduce inflammation [11]. Crimi and colleagues found no correlation between numbers of inflammatory cells and the degree of AHR in humans [80]. Similarly, Wilder reported a dissociation of AHR from immune responses in mice [81]. OVA-sensitization in neonatal mice doubled airway smooth muscle innervation and induced persistent AHR even after inflammation had subsided [10]. Ablated TRPV1-expressing vagal sensory neurons in mice prevented AHR following OVA-sensitization without reducing airway inflammation [11]. However, another study found that ablating sensory neurons both prevented AHR and reduced inflammation [12]. In that study, ~80% of airway nociceptors were silenced, and the authors concluded that inactivating a large population of nocieptor sensory neurons might be required to dampen inflammation. Similarly, mice lacking TRPA1 also had reduced AHR and decreased inflammation [14]. These studies collectively suggest that the degree of AHR does not necessarily correlate with the degree of inflammation, and that many factors, including the initiating event and/or responsible ligands, the type of sensory receptor, and the specific neurons and other cell types expressing the receptor, ultimately determine the relationship between AHR and inflammation.

Previous studies have shown that tachykinin antagonists decrease airway inflammation [76, 77, 82]. Therefore, it is interesting to note the paradoxical increase in IL-13 in the bronchoalveolar lavage fluid of OVA-sensitized *ASIC1a*^*-/-*^ mice despite decreased levels of substance P. A somewhat similar paradox occurs with *ASIC3*^*-/-*^ mice in a murine model of arthritis; *ASIC3*^*-/-*^ mice display a lack of pain despite having greater IL-6 levels [83]. While the mechanisms underlying the elevated IL-13 in the OVA-sensitized *ASIC1a*^*-/-*^ mice are uncertain, it is possible that loss of ASIC1a prevents proton-mediated repression of IL-13 release and/or transcription. It is also possible that ASIC1a expression in dendritic cells [84] or T cells [85] contributes. However, T-cell-mediated cytokine production is not affected by loss of ASIC1a [85]. Therefore, the cell type and mechanisms underlying increased IL-13 levels in OVA-sensitized *ASIC1a*^*-/-*^ mice remain unknown.

These findings suggest that ASICs play a key role in the bronchoconstriction associated with the OVA sensitization model and perhaps with asthma. When combined with previous studies, our results suggest that the acidosis associated with asthma [31–34] may activate ASICs on vagal sensory neurons. Consistent with that suggestion, acid depolarizes vagal nociceptive and mechanosensory airway afferents [28]. The pH reductions induce activity in these afferents, and the currents exhibit kinetics of ASIC channels independent of TRPV1. The activation of vagal neurons may initiate reflex efferent nerve activity and/or may release sensory neuropeptides, including substance P [39] and CGRP [14]. Congruent with that prediction, loss of ASIC1a reduced the substance P concentration in bronchoalveolar lavage liquid; this finding mirrors the finding that loss of the TRPA1 chemosensory receptor decreases bronchoalveolar lavage fluid levels of substance P, CGRP, and neurokinin A [14]. Multiple studies indicate that substance P mediates airway smooth muscle contraction and contributes to AHR [73, 74, 76, 77, 86], although some studies suggest that substance P can relax pre-contracted smooth muscle [87, 88]. Thus, the reduced substance P might, in part, contribute to the reduced AHR in ASIC1a^-/-^ mice.

Even though loss of ASIC1a reduces acid-induced transient currents, we are not aware of any study suggesting that loss of ASIC1a causes a universal elimination of neural activity or universal loss of function. For example, *ASIC1a*^*-/-*^ mice develop secondary paw hyperalgesia in response to carrageenan-induced muscle inflammation [89], and in *ASIC1a*^*-/-*^ mice, paw withdrawal responses to heat are not affected [90]. Thus, it is predicted that the loss of ASIC1a prevents airway hyperreactivity only when the initiating stimulus involves an acidic pH or a ligand that activates ASIC1a.

Our study also has limitations. Although ASICs are present in neurons innervating airways [24, 91], we do not know the identity of the specific neuronal afferents. In addition, we cannot determine the contributions to AHR of ASIC1a in peripheral *vs*. central neurons. It is also possible that ASIC1a might contribute to AHR by modifying mechanosensation [92], and although we could not detect ASIC expression in airway smooth muscle, loss of ASIC1a in non-neuronal cells might also be important [93–95]. Finally, given species differences in innervation of the airway, we are uncertain about whether our observations in *ASIC1a*^*-/-*^ mice will apply to other species.

In summary, our data identify ASIC1a as an important mediator for AHR in OVA-sensitized mice, and suggest that ASICs may play a novel role in the coupling/decoupling of airway inflammation and AHR. In addition, we report for the first time a role for ASIC1a in diminishing the release and/or induction of substance P in inflamed airways. Whether loss of ASIC1a affects concentrations of other sensory neuropeptides remains to be determined. Finally, we speculate that ASIC channel inhibitors might be beneficial in asthma and other airway diseases.
